# Does Humanness Matter? An Ethical Evaluation of Sharing Care Work with Social Robots

**DOI:** 10.1007/s11948-025-00547-y

**Published:** 2025-07-28

**Authors:** Emilian Mihailov, Tenzin Wangmo

**Affiliations:** 1 Research Centre in Applied Ethics, Faculty of Philosophy, University of Bucharest, București, Romania; 2 Institute for Biomedical Ethics, University of Basel, Basel, Switzerland

**Keywords:** Human contact, Robot touch, Humanness, Social robot, Eldercare, Dignity, Dual character concept

## Abstract

While social robots offer potential benefits like task assistance and companionship, their integration raises concerns about the erosion of human connection and the dehumanization of care. Through a qualitative study of older adults, family caregivers, and professional caregivers in Switzerland, we examined their perceptions of social robots and their understanding of the “human contact” in eldercare. Findings revealed the importance of emotional warmth, complex social interactions, and empathy. However, participants also acknowledged the potential benefits of such robots in specific tasks. We argue that the ethical assessment of care robots should focus on determining when robotic contact is desirable. By understanding the limitations of human connection and that humanness is a dual character concept (both descriptive and normative), we identify scenarios where social robots may offer advantages, such as providing care without judging and stimulate social engagement. Robotic “touch” can potentially complement human care in certain situations, preserving older persons’ dignity and improving their quality of life.

## Introduction

The increasing need for caregivers has created opportunities for companies to develop social robots to assist in completing care tasks. One such robot is Pepper, which can move, understand its environment, and communicate with people using body language and speech. It can recognize emotions based on facial expressions and voice tone (Pandey & Gelin, 2018). An emerging strand of research has focused on the institutional, social and psychological factors that facilitate the acceptance of such gerontechnology by older people, caregivers and family members (Huang & Oteng, 2023; Felber et al., 2024). Older people are open to using social robot for different reasons (Vandemeulebroucke et al., 2021; Naneva et al., 2020), including loneliness and the need for verbal interaction (Sorell & Draper, 2014; Vandemeulebroucke et al., 2018; Kuhne et al. 2022, Fracasso 2022).

Even if we suppose that older people are content with being cared for by robots, there is an influential argument that the robotization of eldercare will cut people off from human connectedness (Sparrow & Sparrow, 2006; Parks, 2010; Sharkey & Sharkey, 2012a; Coeckelbergh, 2015; Sparrow, 2016; Vercelli et al., 2018; Boada et al., 2021). The evolution of introducing social robots is considered a natural consequence of technology’s dominancy in society that would lead to dehumanized care practices (Vandemeulebroucke et al., 2020a, b). Physical touch, seeing, and hearing other people are crucial for older adults, especially for those who are more dependent on care and living alone (Sundgren et al., 2020). Hence, social robots should not be acceptable insofar as they take over particular human tasks (Coeckelbergh, 2015). The fear of technology replacing human care is a major factor which drives the appreciation of human connection, as shown by empirical studies (Wu et al., 2014; Wangmo et al., 2019; Continentale, 2019; Sundgren et al., 2020; Boada et al., 2021; Felber et al., 2023). This “humanness” argument highlights that a reduction in human contact could be detrimental to caring practices. As Noel Sharkey and Amanda Sharkey (2012a) put it, “the elderly may find themselves in a barren world of machines, a world of automated care: a factory for the elderly.”

However, there are many cases of neglect in nursing homes and in the community, which shows that just being around other people could be harmful (Yon et al., 2017, 2019). Kitwood (2005) addressed the issue of malignant social psychology to describe how people with dementia are approached and treated by others. If humans are defined by empathy, they are also defined by failures of empathy when they are burdened and stressed. Carers sometimes infantilize, intimidate, label by the name of the disease, invalidate, banish, ignore, and mock those they care for. We should not be surprised that people could be drawn to advertisements for social robots, such as the one stating: *Do not leave your family in the care of a human whose character you know nothing about*.^1^

Further, we may feel embarrassed around others because our body does not conform to fashionable standards. We may value our privacy more than being exposed to interaction. Being around people in potentially vulnerable contexts may hurt our dignity (Felber et al., 2022). In day-to‐day life, older people may often face humiliation for what they do not have anymore: a capacity for self‐governing and independence (Pageau et al., 2024). So, it would be appealing to prudentially avoid stigmatization in human interaction.

In the ethical assessment of social robots, human contact is idealized due to a general tendency to value humanness. Culturally and philosophically, humanness has been associated with moral virtues, empathy, and capacity for recognizing persons and respecting them (Glover, 2012). In this paper, we challenge what seems to be an ethical consensus that reducing human contact is *inherently* bad. We will make the case that humanness is a dual character concept, which helps us understand people’s experiences of rejecting human contact. Dual character concepts are partly descriptive and partly normative, with these dimensions related but quasi-independent (Del Pinal & Reuter, 2017; Knobe et al., 2013; Leslie, 2015). For instance, a father is someone who is biologically related (descriptive dimension), but also someone who should provide love, support and guidance (normative dimension).

We will use empirical data from a qualitative research (Martani et al., 2024; Wangmo et al., 2024) to explore study participants’ understanding and assessment of the “human element” in eldercare. Inspired by the data, we will argue that using assistance from social robot can be justified in certain situations after carefully considering the specific needs and preference of older persons. Social robots can improve human relationships by promoting social engagement or by alleviating caregiver stress and workload, thereby potentially freeing up time for human-to-human interaction (Van Wynsberghe, 2013; Van Kemenade et al. 2015; Ienca et al., 2016; Cifuentes et al., 2020; Hung et al., 2021; Mihailov, 2024; Macalupu et al., 2025; Voinea & Wangmo 2025). First, we argue that a substantial replacement of human beings in the near and distant future is unlikely because caregiving is holistically complex and from the data analzyed from older participants in this study, it appears that they are unwilling to conform to a robotic authority. Second, we argue that social robots can offer better care in certain situations because they lack human traits. Their lack of judgmental tendencies protects the dignity of older persons during personal hygiene and improves care when dealing with intimate health information. Unlike humans, social robots remain free from irritation and anger even when faced with repetitive behaviors, a common and burdensome symptom in dementia patients. A general approach to social robots according to which human contact is always better may not advance the goals of care. Our analysis contributes to how we develop and integrate emerging technologies in eldercare.

### Methods for Empirical-Normative Integration

Empirical data for this paper stem from the project of one of the co-authors (Felber et al., 2024; Martani et al., 2024; Wangmo et al., 2024). It comes from qualitative interviews carried out with 67 individuals (older persons 65 years and older, family caregivers, and professional caregivers) from Switzerland. During the interview, a semi-structured interview guide was used and the participants were posed questions related to different technologies (e.g. wearables, ambient sensors, and robotic technology such as Paro and Pepper). Study participants were recruited using purposive and snowball sampling methods. That is, study information detailing inclusion and exclusion criteria, study purpose were advertised at different venues (e.g. nursing home, assisted living facility), using services that cater to older persons (e.g. meals on wheels), and social media. The inclusion criteria were set broadly as (1) being an older person 65 years and older or being a family or professional caregiver to older people; and (2) living in Switzerland at the time of interview. Thus, we recruited older persons living at home as well as those living in nursing home or assisted living facilities (*n* = 27). The professional caregivers were those supporting older adults in nursing homes, assisted living facilities, or providing home health care (*n* = 23). Family caregivers were unpaid family members who reported that they are providing some care to an older family member (*n* = 17). The interviews were carried out by research assistants at a place and time of participant’s choice. They were on average 96 min (46–189 min) and were transcribed verbatim for data analysis.

From the extensive qualitative data corpus, for this paper, TW extracted all data related to the social robot, Pepper, which was the technology example used as part of the data collection. That is, we sorted all the parts of the interviews from the participants, where they talked about their knowledge and experience of Pepper, whether they will find it useful, and social as well as ethical concerns they see with its use in the care of older persons. TW first coded this dataset limited to Pepper resulting in several themes (e.g. ethically relevant values, acceptance of Pepper). Among the, ethically relevant values, was the concern of replacement of human care and the emphasis placed on the humanness of care, leading to the development of this specific paper critically evaluating the notion of “humanness”. The two authors then read and independently coded all segments from the data, which referred to the idea of human care and the “humanness” of care. In this process, they used applied thematic analysis (Guest et al., 2012). In line with the theoretical arguments presented above, they agreed to present the empirical results within two themes surrounding what social robot Pepper lacks (i.e. Limitation of Pepper) and (b) how it could be beneficial (i.e. Utility of Pepper).

The normative-empirical integration (Wangmo et al., 2023), is based on the method of wide reflective equilibrium, in which data on stakeholder attitudes can have an important role to play in shaping ethical argument but only as potential input into a deliberative process that looks for coherence between attitudes and ethical principles (Daniels, 1979; Savulescu et al., 2021). Older adults’ and caregivers’ moral attitudes towards socially assistive robotics seem to be mainly based on their experiences with aged care and a basic understanding of what socially assistive robotics are and how they can be used (Vandemeulebroucke et al., 2020a, b). This method has been tailored to serve empirical bioethics projects by several scholars (Ives & Draper, 2009; Van Thiel & Van Delden, 2010; de Vries and van Leeuwen, 2010). Wide reflective equilibrium is a two-way dialogue between ethical values and practice (empirical data). The research begins with a conceptual understanding of the ethical issues relevant for the topic, followed by the collection of empirical data based on the ethical concern teased out from conceptual work. In this process, we go back and forth between the normative framework and empirical facts (data available from the study or other sources) until we produce a moral coherence.

When it is not clear how to reach a strict moral coherence we have to balance as far as possible, the range of concerns, interests and priorities that operate on an ethical problem (Ives, 2014). The data we discuss will be integrated in our normative argument as input in the empirical premises and also as a feasibility constraint for general ethical standards. As stated, we will use the methods of reflective equilibrium (Daniels, 1979; Savulescu et al., 2021) to evaluate when empirical data is reliable and when it is worth overriding the data in light of critical reflection.

## Results

### Limitations of Pepper

The study participants evaluated the social robot, Pepper, as *limited in its capabilities to hold complex social interactions* given their care needs. Complex social interactions among others meant the human capability to respond adequately and validate emotions and feelings, understand subtle cues of its users, and maintain open ended conversations. Underlining these deficiencies, a professional caregiver noted: “it’s a machine, (…) it has no feeling, nothing. (…) I don’t really want that, certainly not, but I mean, some…there’s a robot in front of you and you talk…it’s all controlled! There must be emotion, (…) (Professional caregiver 1).” Similarly, an older participant (OP11) stated that Pepper cannot provide the social contact of a family member and “above all, it has no feelings, no human emotions. You can’t implant them.” Along the same lines, participants pointed out that although Pepper has a voice and may be able hold conversations with its user, the above mentioned qualities that it lacks makes it an imperfect imitation of a human person. For example, one professional caregiver (PC6) stated: “Because if you respond and validate, then you are actually in a rapport, in a relationship. And the machine will never be able to build a relationship.”

Another concern was that the *robot is not natural* and humans are: “It [Pepper] is not natural. It’s just that. It [human] is natural - I know what a person looks like, and I don’t know that anymore [with a robot] (Older person 6).” Related was the idea that Pepper is *cold*, whereas humans are warm. Within this naturalness concept was the acceptance that care context is essentially a *relationship* context. Caregivers interviewed in the study felt that such relationship cannot be formed by or with the robot, as it could be with a human.

Family caregiver 1: That when you touch it, it’s cold. (…) Yes (….) It might be fun for people to see how it works, how it moves. But I don’t see it as an interpersonal relationship at all. Not even with the voice [personalized voice of the daughter].

Older person 8: It’s just, it seems to me, it’s a cold thing when a guy like that stands in front of you, it seems to me, it’s a cold thing. It’s a cold thing, is my feeling. Because when you have a person in front of you, it’s something completely different. Because it’s human to human instead of having a robot in front of you. ... It’s much nicer, human to human.

Study participants further noted that Pepper is *a machine* and, as such, it is “dumb” because it could not issue instructions and interpret information beyond what it is made capable of doing. Also the participant acknowledged the ability of robots to perform mechanical tasks, but questioned its capacity to provide care in a way that is comparable to human care. Thus, Pepper does not possess human qualities such as the ability to make decisions (e.g. what do I do first when the boss wants his breakfast) and to be empathetic.

Family caregiver 6: I simply miss the - it’s a tool, it’s mechanical, I simply miss the human element. So I’d rather do without it. So ... There’s a strong tendency to get into the mechanical. And I can imagine that a robot can do certain things, like assembling a car. But in terms of care and support, in human terms, I have trouble with that.

Professional caregiver 9: I think Pepper would think it’s just an aggressive person [talking about person with dementia] and would react the wrong way ... People, so caregivers can show like empathy and stuff ... listen to what it was, even though the resident might keep repeating the same things. But people can, we can just decide, ah yes, they have dementia. And we just have to listen, react a bit and things like that, we can decide that ourselves. (...). But I don’t know how well Pepper can do that like a human, I don’t know.

One participant even rejected the idea of a robotic authority. Though robots can issue commands, probably their status of machines and limited capabilities for social interaction generated the reluctance to follow instructions about personal well-being: “And if it gives me the advice that I should move, then I can still do that, so whether I want to move or not (…) I don’t think I need it for that.” (Older person 3).

### Utility of Pepper

Study participants acknowledged that social robots like Pepper can be beneficial as it can perform *certain tasks*, but it should not be a replacement of human care (because it lacks certain qualities as described in the previous theme). Underscoring fear of replacement of human care, several participants said they cannot imagine eldercare being carried out by robots, “Yes, well, I don’t find it [Pepper] so positive. (…) I just have the feeling that a human being is a social being and needs other people, it doesn’t need a robot (Older person 2).” While others also felt that it is strange, they saw that care by robots may be a possibility: “It’s strange for me at the moment, but I could imagine it (Professional caregiver 2)”.

Therefore, adding to this line of being open to robotic care, a participant pointed out that because of their lacking abilities to judge its user, *some activities might just be better performed by robots than a human*. These included activities that when carried out by robots may enhance the dignity of older persons. For example, an older woman relayed that she would rather by bathed by a robot than different caregivers who help her in this task. She finds uncomfortable that so many caregivers see her in such state of vulnerability. Older adults thus need not feel ashamed when a robot helps them for similar personal tasks. Also noted, but only a very few times was the notion that a functioning robot that is “nice” would be better than an unsympathetic human caregiver or that it is better to have robotic care than no care.

Family caregiver 7: Because these feelings of shame, that’s also something that persists into high dementia, especially for men who have to be washed by women. Yes, the majority of care workers are women. And vice versa, when the mother-in-law needs a shower and the apprentice (male) comes, that’s not an option... From that point of view, it could perhaps also be a support. You just stand in the shower and Pepper lathers your back, why not?

Family caregiver 5: So possibilities, I’ve just said, if he ever needs it…. I think there could be a situation in our care crisis where we, or the generation of our parents and grandparents, would be very happy to receive care from a robot instead of no care at all.

Other services that Pepper can offer followed the general ideas of “*robot as a servant*”. That is, it can perform tasks that do not require much thinking. Just following orders fits again with the concept of unsophisticated robot reported by the participants in the previous theme. An older participant revealed that the robot can take care of household tasks: “I’d like to have a regatta today, do it... Yes, we have laundry today, put it in (Older person 12)”. These tasks including technical chores such as giving medications, bringing food and drinks, which may relieve the care professionals and alleviate their care burden. For example, an older participant stated, “I wouldn’t want the personal things to be handled by a robot like that. I mean like bringing food and tablets, why not?” (Older person 10).

The hope was that the time saved from these technical tasks will then be used to meet the other needs of the older persons. Also noted was that the robots could be conversational partners for those older persons who are lonely to reduce loneliness among this group of older adults in the nursing care setting.

Older person 12: Not only that, but in old people’s homes, every carer is happy if someone is still entertaining people and doing something for them, because they can’t keep up. They’re overworked and don’t have enough people.

Professional caregiver 17: It’s the loneliness, of course, that the old people have, and I realize that every day, that they’re lonely when the family doesn’t have time to come every time, and that’s replaced with a robot. But at least you can see the face of this lady who…yes, that’s the loneliness they have and when a robot like that talks to them and gives them attention. Is valuable to them.

## Discussion

The qualitative data gathered opinions from older persons, family carers, and care professionals about humanness and lack thereof when Pepper was used in elder care. The data on limitations suggests that a significant replacement of human caregivers is unlikely because caregiving is holistically complex and older participants are reluctant to accept robotic authority because they tend to place robots’ utility in spaces that would maintain their own authority over it. Also, participants’ responses on utility of Pepper inspired our argument that social robots can be justified in specific eldercare situations. Crucially, we discuss that social robots can sometimes offer superior care due to their lack of human traits.

### Substantial Replacement of Human Care Is Not Feasible

From the empirical part of our study, we clearly see that our participants, who could be potential future users of the robot Pepper, are skeptical about the possibility that social robots could substantially replace humans in the eldercare space. Current social robots like Pepper lack capabilities to engage in complex social interaction, especially the ability to provide emotional warmth. Furthermore, to some participants, robots (in general) were effective machines for factory work but not suited to surpass humans in caregiving tasks.

By contrast, in the scholarly literature, dystopian scenarios and industrial metaphors (care factories, automated nursing homes) have shaped discussions about social robots, as many fear that a robotization of care will (unintentionally) deteriorate the well-being of the elder population (Sparrow & Sparrow, 2006; Sharkey & Sharkey, 2012a; Coeckelbergh, 2015; Sparrow, 2016). The worry about introducing social robots in eldercare is based on the assumption that humans will be replaced by technology, as it happened in other domains. One reason for this is that strong economic pressures on the care sector will probably lead to less human interaction for older persons (Sparrow & Sparrow, 2006). We should not just assume that any development in social robotics will strictly adhere to their designers’ intentions. Actual robotic performance can deviate in practical applications, and such deviations might be crucial for understanding their impact on eldercare.

However, a scenario in which humans are substantially replaced by social robots in elder care is unlikely to happen in the near future. For our participants, the caring context was deeply relational and, thus, fundamentally different from successful cases of automation like manufacturing. Since the Industrial Revolution, skilled human labor was replaced by the efficiency of machines. Industrial robots that assemble our cars and computers are impressive technological solutions. So, the modern factory has become the paradigm of automation. But manufacturing tasks are fragmented, specialized, and highly standardized. Caring tasks could not be more different. Literature in the field of nursing underscore the multi-skilled and highly contextual work that human caregivers perform to meet the physical, social and emotional needs of their older residents and patients in a *holistic* manner (Smith et al., 2008; Timby, 2009; DeWit & Williams, 2013; Perry et al., 2021). There is a need for daily flexibility in dealing with a wide variety of older persons living in the nursing care institution and the specific care situation at hand. Whereas automation is economically attractive in manufacturing, it may not be efficient in eldercare because there is no final product that must be assembled in conformity with strict standards. Automated care factories are unlikely to prosper in the near future.

Professional caregivers (e.g. nurses) will continue to do most of the care work because caring for people involves many complex skills and tasks that hang together. Their competence expands beyond providing warmth to include a fine-grained grasp of the medical and social context. Even if social robots become very good at specific tasks like setting alarms, providing reminders, and giving medicine, humans will still have to manage all these tasks and work together with other staff members to provide the holistic care of the individual care recipient. When such robots were introduced in Japan, the need for human labor actually increased, rather than decreased (Wright, 2023) since the nurses had to ensure that the technology was working as expected.

Moreover, we observed a potential resistance in our participants to following instructions issued by social robots. Most participants were willing to accept social robots as “servants”, performing technical tasks like doing laundry, bringing food and drinks. Taking over physical labor by such robots relieves the care professionals and help alleviate their care burden. Because of a human resistance to robotic authority, it seems more likely that caring practices will design robots to follow human orders, rather than the other way around. This challenges the idea of social robots *taking over* caregiving roles (Coeckelbergh, 2015).

The complexity of caring tasks and the unwillingness to obey a robotic authority makes it unlikely to reach a substantial replacement of human caregiving. It is more likely to see limited support. As Shannon Vallor has argued, “if carebots provide forms of limited support that draw us further *into* caregiving practices, able to feel more and give more, freed from the fear that we will be crushed by unbearable burdens, then the moral effect on the character of caregivers could be remarkably positive.” (Vallor, 2011, p. 260).

### Differentiating Emotional Neglect from Reduced Human Contact

Two types of arguments have been proposed to justify why human contact is morally important. These arguments are most effective in condemning emotional neglect (which no one disputes), but they do not imply that a reduction of human contact is impermissible. As we will clarify, it is a mistake to equate a reduction of human contact with emotional neglect.

The first kind of argument focuses on human attachment and warmth as an instrumental value in the emotional and cognitive development of human beings (Sparrow, 2016). Human beings are inherently social animals and, so, they rely on communities for survival and flourishing from a young age. The nature of human psychology is such that deprivation of human contact may have dramatic impacts on a person’s well-being and health. Take the example of children who were abandoned in Romanian orphanages during the regime of Nicolae Ceaușescu. No one played with them, held them or talked to them. Severe social and emotional neglect caused these children to exhibit symptoms similar to severe intellectual disability (Gopnik, 2009). The second type of argument is based on interpreting the concept of good care. To evaluate care robots, Coeckelbergh articulates a normative ideal of good care in which he includes the requirement of human contact. Because good care does not only mean physical care but has also psychological and relational dimensions, it “should always involve a significant amount of human contact” (Coeckelbergh, 2015, p. 269). Leaving someone isolated most of the time even if they receive adequate medical assistance, does not constitute good care.

But these arguments do not entail that *any* decrease in human contact is detrimental to older people’s well-being. It only follows that emotional neglect is damaging for social animals like us. This is the case with the Romanian orphanages and the dystopian scenario of an automated nursing home in which older persons never need to deal or talk with a human being. These arguments show only that humans require a certain amount of social interaction in order to thrive. Sparrow (2016) and Coeckelbergh (2015) have not clearly defined what this necessary or significant level of contact is, but they likely refer to a general standard of personal well-being. In fact, these arguments imply that reducing contact is acceptable as long as it does not fall below a general threshold required for well-being. Note also that this threshold is not just about human contact. Some participants suggested that it is dependent on close relationships. Nursing staff can have impersonal contact, which might be inconsequential for ones’ well-being, whereas family caregivers and relatives are the ones who provide a sense of calm and support.

### Humanness Is a Dual Character Concept

Admiring the positive aspects of being human can make us overlook the fact that humans are also responsible for terrible atrocities. This reminder motivates us to question whether human contact is *always* preferable to contact provided by a social robot. To understand the puzzle that humanness can be both good and bad, we will make the case that it is dual character concept (Del Pinal & Reuter, 2017; Knobe et al., 2013; Leslie, 2015). Take the concept of a painter. A person is a painter if descriptive aspects specific to their activity are met, such as painting pictures for a living. On the other hand, a person is a painter if they are committed to the norm of painting well-crafted pictures. So, people can categorize a person as a painter either descriptively or normatively.

Similarly, the concept of humanness has both descriptive and normative dimensions. What make us human are descriptive aspects specific to our psychology, as well as the values that we uphold. Not everything that makes us (descriptively) human is morally desirable. Indeed, what defines human beings are empathy and sophisticated cooperation and these can be morally endorsed, but we also have a tendency to engage in strategic and proactive violence more than any other species (Henrich, 2016; Wrangham, 2019). While a propensity for proactive violence is a descriptive trait of the human species, it is not necessarily a normative ideal. Moreover, descriptive human traits have a dual use. They are beneficial in some contexts and toxic in other contexts. Tribalism, understood as group bonding and fusion, is specific to human beings. A strong connection with a group identity has motivated many peaceful forms of cooperation, but it also has produced some of the cruelest atrocities (Whitehouse, 2024).

Once we understand the dual character of humanness, it becomes clear that it is insufficient to state that social robots lead to a reduction in human contact. We still have to argue whether a specific human contact is morally desirable. Thus, we need a nuanced approach about the value of human connection. For one thing, increasing social interaction can even be detrimental if it does not consider individual needs and preferences. Older adults are a heterogenous group and, consequently, their social engagement preferences varies. Some elder patients need more distance and do not accept affective touch from nursing professionals, while others find hugging natural (Długołęcka et al., 2024). Patients with reduced physical function prefer social engagement within the nursing facility, rather than outside (Jang et al., 2014). While older persons have a strong preference for their migrant caregivers to understand them as individuals, they show little interest in sharing social or leisure activities with their caregivers (Cohen-Mansfield et al., 2019).

### The Case for Robot “touch”

Human contact is not the only source of connection. Some participants suggested that there are activities, which might be better performed by robots than humans because they lack human traits. Older individuals, particularly men, might feel less embarrassed if personal care tasks were performed by a social robot instead of a female care provider because they often experience shame when needing assistance with intimate tasks. Discomfort can also arise in situations where a young male caregiver assists an older women as well as in situations where one is supported by several caregivers for intimate tasks, as delineated by a study participant.

#### Robots don’t judge, Humans do

A sense of personal dignity is relevant for the day-to-day experiences of the older persons (Nordenfelt, 2004; Ternestedt, 2009; Barclay, 2016; Pageau et al., 2024). Research on these experiences has revealed a connection between loss of dignity, shame and humiliation (Killmister, 2010). Older patients with dirty clothing who are not cleaned in due time may appear in the eyes of others as objects of pity and less than exemplary human beings (Barclay, 2016). Moreover, they may feel humiliation and shame when their privacy and norms of decorum are not respected (Gallagher et al., 2008; Baillie, 2009; Guo & Jacelon, 2014). Older residents with dementia may sometimes be left on the toilet with the bathroom door open while the caregivers attend to other tasks. When residents make requests about discomfort, the caregivers can be impatient and speak to them in a condescending manner (Barclay, 2018).

Human beings are strongly inclined to categorize others based on smell, appearance, speech, and behavior, to decide how they fit in socially (Berreby, 2008). This makes us the most judgmental species. In contrast, social robots can be designed to lack these human biases. They can provide services without judgment. They can offer patient assistance, regardless of a person’s physical appearance or gender or limitations. Because it lacks human schemas of interpretation, social robot’s involvement can enhance the dignity of older persons in contexts of personal hygiene (Felber et al., 2022). Shame is often caused by the human-to-human element of care and this can stop them from getting necessary care and can also prevent them from sharing important health information (Palmer & Schwan 2022). Disclosure of personal information is often low due to embarrassment. In a study about people’s willingness to disclose sensitive information, participants who were interviewed by a faceless avatar reported more information of higher quality about a personal event than did participants who were interviewed by a human-appearing avatar (Hsu et al., 2024). This illustrates how the human element may become an obstacle when it is concerned with sharing intimate health information.

Older persons may feel less embarrassed and less dependent on humans if they had the option to interact with a social robot. Wachsmuth (2018) documents undignified experiences born from human connection: “I would, in old age, rather have a robot help me to get dressed than show myself naked in front of a nurse.”; (a 75 year old): “I might prefer a feeding robot over a nurse since I would be in control when the spoon comes and not depend on the caregiver.”; “I would not be a burden on a robot.” Wachsmuth recounts how his 92-year-old aunt became confined to her bed in a nursing home and needed to wear diapers. On the phone she said to him: “Now they took my dignity, I have to wait to get my diapers changed.”

We should not expect caregivers to do everything all at once. They are human beings who get exhausted, irritated, and angry at those who they care for. They can emotionally reject elder persons when they are overloaded. Caregivers often experience physical strain from tasks like supporting, lifting, and transferring patients from their wheelchairs to a toilet or helping patients enter and exit bathtubs. By reducing the burden of physically demanding tasks (Homma et al. 2009; Satoh et al. 2009), technology like social robots has the potential to prevent nurse burnout, irritability, and potential neglect of older persons living in nursing homes or receiving nursing care while living in the community. Similar benefits can happen in distributing caring tasks to social robots as suggested in the empirical findings.

#### Conversational Robots are Good Listeners

The relationship between care robots and tasks performed by humans is not a zero-sum relationship. Human activity can integrate assistive technology in a mutually enhancing way (Mihailov, 2024). Generative AI is impressive in how well it simulates empathy, compassion, and perspective-taking to support patients in spite of the machine’s coldness (Inzlicht et al. 2023). Though it is controversial what emotions should be expressed in human-robot interactions (Fosch Villaronga, 2019), AI agents can communicate with humans in ways that makes them feel heard (Yin et al., 2024). If one is too worried about an attempt to substitute robot simulacra for genuine feelings (Sparrow & Sparrow, 2006; Segers, 2022), human nurses sometimes “fake” emotional care (because of personal reasons) or at least act professionally without emotional investment, yet their patients still feel adequately cared for (Lancaster, 2019). Introducing social robots as conversational companions can shift the focus of human conversation. Instead of reducing overall communication, it might redirect it towards other goals, such as strengthening relationships with family or team members. This can ultimately improve a person’s ability to effectively communicate. Sharing conversational activities with social robots add up overall, creating more opportunities for social connection, learning activities, and emotion expression. There is evidence that interactions between social robots and older persons in care homes increased the social interactions between the residents themselves and made taciturn residents more talkative with family members and nursing staff (Wada & Shibata 2006).

Most importantly, social robots unlike humans maintain focus and lack irritation, even when faced with repetitive queries as it often happens in patients with dementia. Repetitive behaviors refers to responses characterized by their repetition, inflexibility, frequent lack of obvious function, and inappropriateness (Lewis & Kim, 2009). These behaviors are common in dementia patients and considered the most burdensome symptoms, being correlated with a reduced quality of life for caregivers (Coen et al., 2001). Repetitive behaviors can be disruptive and overwhelming for those who provide care (Cipriani et al., 2013). When caregivers are exhausted by the patients’ repetitive behavior, they may want to avoid the interaction. Thus, using social robots as caregivers for specific tasks, like listening to repetitive talk, could protect older adults from neglect due to the irritable situation and improve the caregiver’s quality of life.

### Limitations

The empirical data used for this study is part of an overall qualitative exploratory research project (Felber et al., 2024; Martani et al., 2024; Wangmo et al., 2024). As such, we cannot claim representativeness of our findings since they are limited to one German-speaking part of Switzerland, study participants were recruited purposively, and social desirability would have influenced their responses. Therefore, the findings are not generalizable. Most importantly, we presented a very specific segment and theme related to a particular social robot studied in the project, which were analyzed by the authors from their own subjective standpoints. We relied on the example of a social robot, Pepper, for evaluating robotic qualities. Consequently, some identified issues and participant observations may be specific to Pepper’s capabilities and limitations, rather than across all potential social robots (e.g. Paro, Aibo).

Finally, though reflective equilibrium is a practical method of integrating empirical data and normative analysis, the iterative process lacks precise guidelines. Researchers often struggle to clearly determine how they balance out normative frameworks and data on moral intuitions and attitudes (Arras, 2009; Paulo, 2020). The iterative process of going back-and-forth between the normative and the empirical to come to a coherent account is fraught with indeterminate indications (Wangmo et al., 2023).

## Conclusion: Bringing Life to Eldercare Institutions

Some of our participants felt that social robots, like Pepper, lacked the warmth that humans have. They highlighted the physical aspect of human touch, unlike the cold sensation of touching a robot. These reactions are understandable. Initial contact with robots can make people reticent due to the cold sensation and absence of a familiar human corporeal connection, but social robots may potentially show behavioral altruism and act in the interest of their patients. Underneath human coldness, there is self-interest and manipulation, while institutional coldness induces indifference towards people’s needs. A neutral coldness that fails to satisfy a desire is preferable to a morally corrupt coldness, which is prone to neglect others.

To meet the challenge of caring for an aging population we have to go beyond human vs. artificial interactions. Institutional dehumanization is a greater danger. What ultimately matters in eldercare is creating spaces in which people feel part of a community and a home, where medicine and institutions do not take control of their lives in old age (Gawande, 2014; Wright, 2023). Robots will not solve the care crisis, yet we need moral imagination to carefully experiment and evaluate how robots can fit into human caregiving in a meaningful way. This requires finding creative ways to improve the quality of life of older people and retraining care staff.

Gawande (2014) tells the story of a medical director who wanted to revitalize a nursing home. Every time he went to work he observed a “institutionalized absence of life”. The nurses told him that this is how things work and would become acceptable over time. He successfully applied for innovation funding and managed to put green plants in every room, created a vegetable and flower garden and brought in birds and dogs. One night at 3:00 a.m. a nurse calls him to say: “The dog pooped on the floor. Are you coming to clean it up?” The nursing school did not prepare her to deal with normal animal behavior. She placed a chair over the poop, and left.

Nursing staff will need to view robots as valuable team members. Surely, they will likely impose new work on both nursing staff and residents, almost as if they were residents themselves (Wright, 2023). But from this position social robots can also bring life in human institutions.

## Data Availability

Anonymized interview transcripts where participants consented to sharing them on an Open Access Platform, are available on SwissUBase.

## Footnotes

1 This advertisement is inspired by Ted Chiang’ sci-fi story, *Dacey’s Patent Automatic Nanny* (Chiang, 2019, p. 176).

## References

[CR1] Arras, J. D. (2009). The way we reason now: Reflective equilibrium in bioethics. In B. Steinbock (Ed.), *The Oxford handbook of bioethics*. Oxford University Press.

[CR2] Baillie, L. (2009). Patient dignity in an acute hospital setting: A case study. *International Journal of Nursing Studies*, *46*(1), 23–37. 18790477 10.1016/j.ijnurstu.2008.08.003

[CR4] Barclay, L. (2016). In sickness and in dignity: A philosophical account of the meaning of dignity in health care. *International Journal of Nursing Studies*, *61*, 136–141. 27351830 10.1016/j.ijnurstu.2016.06.010

[CR5] Barclay, L. (2018). Dignitarian medical ethics. *Journal of Medical Ethics*, *44*(1), 62–67. 29030395 10.1136/medethics-2017-104467

[CR6] Berreby, D. (2008). *Us and them: The science of identity*. University of Chicago Press.

[CR7] Boada, J. P., Maestre, B. R., & Genís, C. T. (2021). The ethical issues of social assistive robotics: A critical literature review. *Technology in Society*, *67*, 101726.

[CR9] Chiang, T. (2019). *Exhalation*. Pan Macmillan.

[CR8] Cifuentes, C. A., Pinto, M. J., Céspedes, N., & Múnera, M. (2020). Social robots in therapy and care. *Current Robotics Reports*, *1*, 59–74.

[CR13] Cipriani, G., Vedovello, M., Ulivi, M., Nuti, A., & Lucetti, C. (2013). Repetitive and stereotypic phenomena and dementia. *American Journal of Alzheimer’s Disease & Other Dementias*, *28*(3), 223–227. 10.1177/1533317513481094 PMC10852852 23512997

[CR10] Coeckelbergh, M. (2015). Artificial agents, good care, and modernity. *Theoretical Medicine and Bioethics*, *36*, 265–277. 26002636 10.1007/s11017-015-9331-y

[CR12] Coen, R. F., Duane, Y., Coakley, D., & Lawlor, B. A. (2001). Behavioural symptoms in alzheimer’s disease: Evaluation of frequency of occurrence and caregiver tolerability. *Irish Journal of Medical Science*, *170*(suppl. 3), 98.11491060

[CR11] Cohen-Mansfield, J., Golander, H., Iecovich, E., & Jensen, B. (2019). Social engagement care for frail older persons: Desire for it and provision by live-in migrant caregivers. *The Journals of Gerontology: Series B*, *74*(6), 1062–1071. 10.1093/geronb/gbx052 28475774

[CR14] Continentale (2019). Welche Chancen und Risiken sehen Sie darin, wenn Roboter Teile der medizinischen Versorgung zu Hause übernehmen würden? Available from: https://de.statista.com/statistik/daten/studie/1065313/umfrage/chancen-und-risike n-durch-roboter-in-der-haeuslichen-versorgung/

[CR16] Daniels, N. (1979). Wide reflective equilibrium and theory acceptance in ethics. *The Journal of Philosophy*, *76*(5), 256–282.

[CR17] De Vries, M., & Van Leeuwen, E. (2010). Reflective equilibrium and empirical data: Third person moral experiences in empirical medical ethics. *Bioethics*, *24*(9), 490–498. 19438441 10.1111/j.1467-8519.2009.01721.x

[CR18] Del Pinal, G., & Reuter, K. (2017). Dual character concepts in social cognition: Commitments and the normative dimension of conceptual representation. *Cognitive Science*, *41*, 477–501. 27859536 10.1111/cogs.12456

[CR19] DeWit, S. C., & Williams, P. A. (2013). *Fundamental concepts and skills for nursing*. Elsevier Health Sciences.

[CR20] Długołęcka, A., Jagodzińska, M., Bober, W. J., & Przyłuska-Fiszer, A. (2024). Ethics of a physiotherapist: Touch, corporeality, Intimacy—Based on the experience of elderly patients. *Journal of Bioethical Inquiry*, *21*, 461–474. 10.1007/s11673-023-10323-x PMC11652398 38748337

[CR22] Felber, N. A., Pageau, F., McLean, A., & Wangmo, T. (2022). The concept of social dignity as a yardstick to delimit ethical use of robotic assistance in the care of older persons. *Medicine Health Care and Philosophy*, *25*, 99–110. 10.1007/s11019-021-10054-z PMC8614079 34822097

[CR23] Felber, N. A., Tian, Y. J., Pageau, F., Elger, B. S., & Wangmo, T. (2023). Mapping ethical issues in the use of smart home health technologies to care for older persons: A systematic review. *BMC Medical Ethics*, *24*(1), 24. 36991423 10.1186/s12910-023-00898-w PMC10061702

[CR21] Felber, N. A., Lipworth, W., Tian, Y. J., Schwab, R., D., & Wangmo, T. (2024). Informing existing technology acceptance models: A qualitative study with older persons and caregivers. *European Journal of Ageing*, *21*(1), 12. 38551677 10.1007/s10433-024-00801-5 PMC10980672

[CR24] Fosch Villaronga, E. (2019). I love you, said the robot: Boundaries of the use of emotions in human-R robot interactions. *Emotional Design in Human-Robot Interaction: Theory, Methods and Applications*, 93–110.

[CR25] Fracasso, F., Buchweitz, L., Theil, A., Cesta, A., & Korn, O. (2022). Social robots acceptance and marketability in Italy and Germany: A cross-national study focusing on assisted living for older adults. *International Journal of Social Robotics*, *14*(6), 1463–1480.

[CR26] Gallagher, A., Li, S., Wainwright, P., Jones, I. R., & Lee, D. (2008). Dignity in the care of older people–a review of the theoretical and empirical literature. *BMC Nursing*, *7*, 1–12. 18620561 10.1186/1472-6955-7-11 PMC2483981

[CR28] Gawande, A. (2014). *Being mortal: Illness, medicine and what matters in the end*. Profile Books. 10.1080/13648470.2015.1096603 27454565

[CR29] Glover, J. (2012). *Humanity: A moral history of the twentieth century*. Yale University Press.

[CR30] Gopnik, A. (2009). *The philosophical baby: What children’s minds tell us about truth, love & the meaning of life*. Random House.

[CR31] Guest, G., MacQueen, K., & Namey, E. (2012). *Applied thematic analysis*. Sage Publications, Inc. 10.4135/9781483384436

[CR32] Guo, Q., & Jacelon, C. S. (2014). An integrative review of dignity in end-of-life care. *Palliative Medicine*, *28*(7), 931–940. 24685648 10.1177/0269216314528399

[CR33] Henrich, J. (2016). *The secret of our success: How culture is driving human evolution, domesticating our species, and making us smarter*. Princeton University Press.

[CR34] Homma, K., Yamada, Y., Matsumoto, O., Ono, E., Lee, S., Horimoto, M., & Shiozawa, S. (2009). A proposal of a method to reduce burden of excretion care using robot technology. In *2009 IEEE international conference on rehabilitation robotics* (pp. 621–625). IEEE.

[CR35] Hoppe, J. A., Tuisku, O., Johansson-Pajala, R. M., Pekkarinen, S., Hennala, L., Gustafsson, C., & Thommes, K. (2023). When do individuals choose care robots over a human caregiver? Insights from a laboratory experiment on choices under uncertainty. *Computers in Human Behavior Reports*, *9*, 100258.

[CR36] Hsu, C. W., Gross, J., & Hayne, H. (2024). The avatar face-off: A face (less) avatar facilitates adults’ reports of personal events. *Behaviour & Information Technology*, *43*(4), 800–810.

[CR37] Huang, G., & Oteng, S. A. (2023). Gerontechnology for better elderly care and life quality: A systematic literature review. *European Journal of Ageing*, *20*(1), 27. 37347277 10.1007/s10433-023-00776-9 PMC10287881

[CR38] Hung, L., Gregorio, M., Mann, J., Wallsworth, C., Horne, N., Berndt, A., & Chaudhury, H. (2021). Exploring the perceptions of people with dementia about the social robot PARO in a hospital setting. *Dementia*, *20*(2), 485–504. 31822130 10.1177/1471301219894141 PMC7983329

[CR39] Ienca, M., Jotterand, F., Vică, C., & Elger, B. (2016). Social and assistive robotics in dementia care: Ethical recommendations for research and practice. *International Journal of Social Robotics*, *8*, 565–573.

[CR40] Inzlicht, M., Cameron, C. D., D’Cruz, J., & Bloom, P. (2023). In praise of empathic AI. *Trends in Cognitive Sciences*. 10.1016/j.tics.2023.12.003 38160068

[CR42] Ives, J. (2014). A method of reflexive balancing in a pragmatic, interdisciplinary and reflexive bioethics. *Bioethics*, *28*(6), 302–312. 23444909 10.1111/bioe.12018

[CR41] Ives, J., & Draper, H. (2009). Appropriate methodologies for empirical bioethics: It’s all relative. *Bioethics*, *23*(4), 249–258. 19338525 10.1111/j.1467-8519.2009.01715.x

[CR43] Jang, Y., Park, N. S., Dominguez, D. D., & Molinari, V. (2014). Social engagement in older residents of assisted living facilities. *Aging & Mental Health*, *18*(5), 642–647. 24345086 10.1080/13607863.2013.866634

[CR44] Killmister, S. (2010). Dignity: Not such a useless concept. *Journal of Medical Ethics*, *36*(3), 160–164. 20211996 10.1136/jme.2009.031393

[CR45] Kitwood, T. (2005). Malignant social psychology. In V. Bacigalupa et al. (Eds)*,**Understanding care, welfare and community* (pp. 225–231). Routledge.

[CR46] Knobe, J., Prasada, S., & Newman, G. E. (2013). Dual character concepts and the normative dimension of conceptual representation. *Cognition*, *127*(2), 242–257. 23454798 10.1016/j.cognition.2013.01.005

[CR47] Kühne, K., Jeglinski-Mende, M. A., Fischer, M. H., & Zhou, Y. (2022). Social robot–Jack of all trades? *Paladyn Journal of Behavioral Robotics*, *13*(1), 1–22.

[CR48] Lancaster, K. (2019). The robotic touch: Why there is no good reason to prefer human nurses to carebots. *Philosophy in the Contemporary World*, *25*(2), 88–109.

[CR49] Leslie, S. J. (2015). ‘Hillary Clinton is the only man in the Obama Administration’: Dual character concepts, generics, and gender. *Analytic Philosophy*, *56*(2).

[CR50] Lewis, M., & Kim, S. J. (2009). The pathophysiology of restricted repetitive behavior. *Journal of Neurodevelopmental Disorders*, *1*, 114–132. 21547711 10.1007/s11689-009-9019-6 PMC3090677

[CR51] Macalupu, V., Miller, E., Martin, L., & Caldwell, G. (2025). Human–robot interactions and experiences of staff and service robots in aged care. *Scientific Reports*, *15*(1), 2495. 39833203 10.1038/s41598-025-86255-w PMC11747507

[CR52] Martani, A., Tian, Y. J. A., Felber, N., & Wangmo, T. (2025). Gerontechnologies, ethics, and care phases: Secondary analysis of qualitative interviews. *Nursing Ethics*, 32, 141–155. 10.1177/09697330241238340 PMC11771086 38470960

[CR53] Mihailov, E. (2024). Is the house of smart technologies less human? Challenges to human dignity in the care of older persons. *Robots and Gadgets: Aging at Home*, 119.

[CR54] Naneva, S., Sarda Gou, M., Webb, T. L., & Prescott, T. J. (2020). A systematic review of attitudes, anxiety, acceptance, and trust towards social robots. *International Journal of Social Robotics*, *12*(6), 1179–1201.

[CR55] Nordenfelt, L. (2004). The varieties of dignity. *Health Care Analysis*, *12*, 69–81. 15487812 10.1023/B:HCAN.0000041183.78435.4b

[CR56] Pageau, F., Fiasse, G., Nordenfelt, L., & Mihailov, E. (2024). Care of the older person and the value of human dignity. *Bioethics*, *38*(1), 44–51. 38073573 10.1111/bioe.13251

[CR57] Palmer, A., & Schwan, D. (2022). Beneficent dehumanization: Employing artificial intelligence and carebots to mitigate shame-induced barriers to medical care. *Bioethics*, *36*(2), 187–193. 34942057 10.1111/bioe.12986

[CR58] Pandey, A. K., & Gelin, R. (2018). A mass-produced sociable humanoid robot: Pepper: The first machine of its kind. *IEEE Robotics & Automation Magazine*, *25*(3), 40–48.

[CR59] Parks, J. A. (2010). Lifting the burden of women’s care work: Should robots replace the human touch? *Hypatia*, *25*(1), 100–120.

[CR60] Paulo, N. (2020). The unreliable intuitions objection against reflective equilibrium. *The Journal of Ethics*, *24*(3), 333–353.

[CR61] Perry, A. G., Potter, P. A., Ostendorf, W. R., & Laplante, N. (2021). *Clinical nursing skills and Techniques-E-Book: Clinical nursing skills and Techniques-E-Book*. Elsevier Health Sciences.

[CR62] Satoh, H., Kawabata, T., & Sankai, Y. (2009). Bathing care assistance with robot suit HAL. In *2009 IEEE international conference on robotics and biomimetics (ROBIO)* (pp. 498–503). IEEE.

[CR63] Savulescu, J., Gyngell, C., & Kahane, G. (2021). Collective reflective equilibrium in practice (CREP) and controversial novel technologies. *Bioethics*, *35*(7), 652–663. 33945162 10.1111/bioe.12869 PMC8581760

[CR64] Segers, S. (2022). Care robots and the value of veracity: Disruption or entrenchment? In *Machines of change: Robots, AI and value change*.

[CR65] Sharkey, A., & Sharkey, N. (2012a). Granny and the robots: Ethical issues in robot care for the elderly. *Ethics and Information Technology*, *14*, 27–40.

[CR66] Sharkey, N., & Sharkey, A. (2012b). The eldercare factory. *Gerontology*, *58*(3), 282–288. 21952502 10.1159/000329483

[CR67] Smith, S. F., Duell, D., Martin, B. C., Gonzalez, L., & Aebersold, M. (2008). *Clinical nursing skills: Basic to advanced skills* (p. 1456). Pearson Prentice Hall.

[CR68] Sorell, T., & Draper, H. (2014). Robot carers, ethics, and older people. *Ethics and Information Technology*, *16*, 183–195.

[CR70] Sparrow, R. (2016). Robots in aged care: A dystopian future? *AI & Society*, *31*, 445–454.

[CR71] Sparrow, R., & Sparrow, L. (2006). In the hands of machines? The future of aged care. *Minds and Machines*, *16*, 141–161.

[CR72] Sundgren, S., Stolt, M., & Suhonen, R. (2020). Ethical issues related to the use of gerontechnology in older people care: A scoping review. *Nursing Ethics*, *27*(1), 88–103. 31113266 10.1177/0969733019845132

[CR73] Ternestedt, B. M. (2009). A dignified death and identity-promoting care. In L. Nordenfelt (Ed), *Dignity in care for older people*, (pp. 146–167).

[CR74] Timby, B. K. (2009). *Fundamental nursing skills and concepts*. Lippincott Williams & Wilkins.

[CR75] Van Kemenade, M. A., Konijn, E. A., & Hoorn, J. F. (2015). Robots humanize care. In *Proceedings of the international joint conference on biomedical engineering systems and technologies* (Vol. 5, No. 1, pp. 648–653).

[CR76] Van Thiel, G. J., & Van Delden, J. J. (2010). Reflective equilibrium as a normative empirical model. *Ethical Perspectives*, *17*(2), 183–202.

[CR81] Van Wynsberghe, A. (2013). A method for integrating ethics into the design of robots. *Industrial Robot: an International Journal*, *40*(5), 433–440.

[CR77] Vandemeulebroucke, T., de Casterlé, B. D., & Gastmans, C. (2018). How do older adults experience and perceive socially assistive robots in aged care: A systematic review of qualitative evidence. *Aging & Mental Health*, *22*(2), 149–167. 28282732 10.1080/13607863.2017.1286455

[CR78] Vandemeulebroucke, T., de Casterlé, B. D., & Gastmans, C. (2020a). Ethics of socially assistive robots in aged-care settings: A socio-historical contextualisation. *Journal of Medical Ethics*, *46*(2), 128–136. 31818967 10.1136/medethics-2019-105615

[CR79] Vandemeulebroucke, T., Dierckx de Casterlé, B., Welbergen, L., Massart, M., & Gastmans, C. (2020b). The ethics of socially assistive robots in aged care. A focus group study with older adults in Flanders, Belgium. *The Journals of Gerontology: Series B*, *75*(9), 1996–2007. 10.1093/geronb/gbz070 31131848

[CR80] Vandemeulebroucke, T., Dzi, K., & Gastmans, C. (2021). Older adults’ experiences with and perceptions of the use of socially assistive robots in aged care: A systematic review of quantitative evidence. *Archives of Gerontology and Geriatrics*, *95*, 104399. 33813208 10.1016/j.archger.2021.104399

[CR82] Vercelli, A., Rainero, I., Ciferri, L., Boido, M., & Pirri, F. (2018). Robots in elderly care. *DigitCult-Scientific Journal on Digital Cultures*, *2*(2), 37–50.

[CR83] Voinea, C., & Wangmo, T. (2024). Socially assistive robots and meaningful work: The case of elder care. *Available at SSRN 4907014*. Advance online publication. 10.1057/s41599-025-05498-0 PMC12254036 40657426

[CR84] Wachsmuth, I. (2018). Robots like me: Challenges and ethical issues in aged care. *Frontiers in Psychology*, *9*, 432. 29666601 10.3389/fpsyg.2018.00432 PMC5892289

[CR85] Wada, K., & Shibata, T. (2006, May). Robot therapy in a care house-its sociopsychological and physiological effects on the residents. In *Proceedings 2006 IEEE international conference on robotics and automation, 2006. (ICRA 2006).* (pp. 3966–3971). IEEE.

[CR87] Wangmo, T., Lipps, M., Kressig, R. W., & Ienca, M. (2019). Ethical concerns with the use of intelligent assistive technology: Findings from a qualitative study with professional stakeholders. *BMC Medical Ethics*, *20*, 1–11. 31856798 10.1186/s12910-019-0437-z PMC6924051

[CR88] Wangmo, T., Provoost, V., & Mihailov, E. (2023). The vagueness of integrating the empirical and the normative: Researchers’ views on doing empirical bioethics. *Journal of Bioethical Inquiry*, *21*, 295–308. 10.1007/s11673-023-10286-z PMC11288993 37938498

[CR86] Wangmo, T., Duong, V., Felber, N. A., Tian, Y. J., & Mihailov, E. (2024). No playing around with robots? Ambivalent attitudes toward the use of Paro in elder care. *Nursing Inquiry*, *31*, e12645. 10.1111/nin.12645 38812242

[CR89] Whitehouse, H. (2024). *Inheritance: The evolutionary origins of the modern world*. Harvard University Press.

[CR90] Wrangham, R. (2019). *The goodness paradox: The strange relationship between virtue and violence in human evolution*. Vintage.

[CR91] Wright, J. (2023). *Robots won’t save Japan: An ethnography of eldercare automation*. Cornell University Press.

[CR92] Wu, Y. H., Wrobel, J., Cornuet, M., Kerhervé, H., Damné, S., & Rigaud, A. S. (2014). Acceptance of an assistive robot in older adults: A mixed-method study of human–robot interaction over a 1-month period in the living lab setting. *Clinical Interventions in Aging*, *9*, 801. 24855349 10.2147/CIA.S56435 PMC4020879

[CR93] Yin, Y., Jia, N., & Wakslak, C. J. (2024). AI can help people feel heard, but an AI label diminishes this impact. *Proceedings of the National Academy of Sciences*, *121*(14), e2319112121. 10.1073/pnas.2319112121 PMC10998586 38551835

[CR94] Yon, Y., Mikton, C. R., Gassoumis, Z. D., & Wilber, K. H. (2017). Elder abuse prevalence in community settings: A systematic review and meta-analysis. *The Lancet Global Health*, *5*(2), e147–e156. 28104184 10.1016/S2214-109X(17)30006-2

[CR95] Yon, Y., Ramiro-Gonzalez, M., Mikton, C. R., Huber, M., & Sethi, D. (2019). The prevalence of elder abuse in institutional settings: A systematic review and meta-analysis. *European Journal of Public Health*, *29*(1), 58–67. 29878101 10.1093/eurpub/cky093 PMC6359898

